# A comparison of dual-bolus and dual-sequence quantitative myocardial perfusion techniques

**DOI:** 10.1186/1532-429X-17-S1-P50

**Published:** 2015-02-03

**Authors:** John D Biglands, David P Ripley, David A Broadbent, David M Higgins, Peter P Swoboda, Adam K McDiarmid, Pankaj Garg, Sven Plein, David L Buckley

**Affiliations:** 1 Division of Medical Physics, Leeds Institute of Cardiovascular and Metabolic Medicine, University of Leeds, Leeds, UK; 2 Department of Medical Physics and Engineering, Leeds Teaching Hospitals NHS Trust, Leeds, UK; 3 Multidisciplinary Cardiovascular Research Centre & Leeds Institute for Cardiovascular and Metabolic Medicine, University of Leeds, Leeds, UK; 4 Philips Healthcare, Guildford, UK

## Background

Single bolus quantitative myocardial perfusion is sensitive to systematic errors due to the non-linear relationship between contrast agent concentration and MR signal. These errors can be minimised by using a low dose contrast bolus or by altering the imaging sequence parameters. However, these approaches substantially reduce the contrast to noise ratio of the images increasing the noise in the myocardial blood flow (MBF) values. This study compared two techniques proposed to address these issues; the dual-bolus technique and dual-sequence imaging.

## Methods

Seven patients undergoing outpatient investigation for suspected coronary artery disease underwent rest and adenosine induced stress dynamic contrast enhanced myocardial perfusion CMR. A pre-bolus of 0.005mmol/kg of Gadovist® was used followed by a main bolus of 0.05mmol/kg. Injection volumes and rates were matched. Imaging was performed using a saturation-recovery prepared turbo gradient echo (SR-TGE) sequence to acquire three slices through the heart (144x144 acquisition matrix, FOV 360x360) with a pre-pulse delay (PPD) of 95.9ms. The Philips instantaneous scan-switching/interleaving capability was used to acquire an additional low resolution (64x64 acquisition matrix) image directly after the first-slice saturation pulse with a PPD of 24.3ms. Pre-contrast proton density weighted (PDW) images were acquired using the same pulse sequence with the saturation pulse turned off. Enhancement curves were converted to concentrations using the pulse sequence equation and parameters in addition to the pre-contrast signal and PDW values. Curves were selected for the two analyses as shown in the figure and the area under the curve (AUC) of each AIF was calculated. MBF values were generated using Fermi-constrained deconvolution.

**Figure 1:**
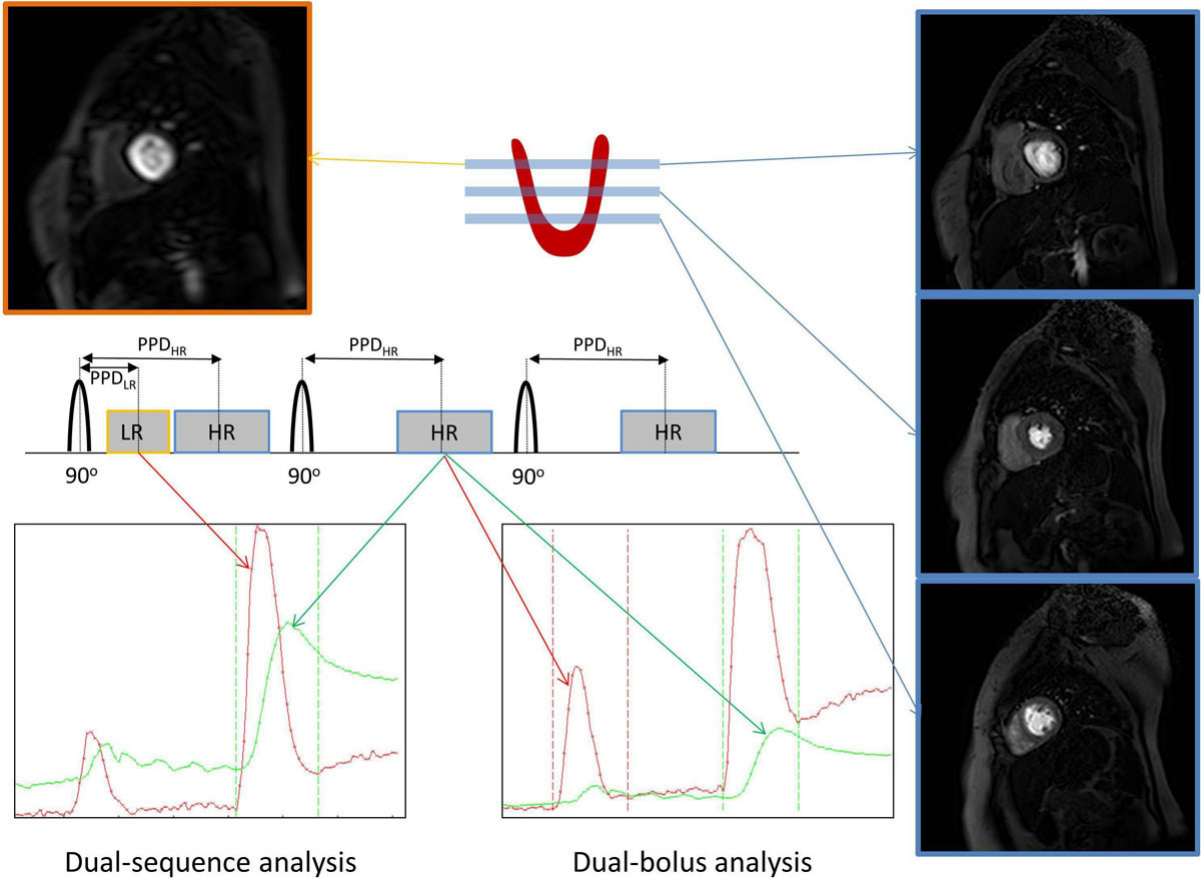
**SR-FFE dual sequence perfusion acquisition.** The low resolution (LR) image uses a shared pre-pulse with the first of three high resolution (HR) acquisitions. Dual-bolus analysis uses the pre-bolus (dose compensated i.e. x10) from the HR images for the arterial input function (AIF) and the main bolus from the HR images for the myocardium. Dual-sequence uses the main bolus from the LR images for the AIF and the main bolus from the HR images for the myocardium.

## Results

The quantitative perfusion results are summarised in the table. Dual-bolus MBF values were significantly smaller than dual-sequence values (p<0.05) and gave a greater MPR (borderline significance p=0.06). The AUC of the dose corrected pre-bolus was significantly greater than that for the bolus (p<0.05).

**Table 1:** Mean and standard deviation MBF values at stress and rest and MPR values (stress MBF/rest MBF) from the dual-bolus and dual-sequence analysis methods.

	Stress MBF (ml/g/min)	Rest MBF (ml/g/min)	MPR
Dual-bolus	1.45 +/- 0.45	0.48 +/- 0.20	3.38 +/- 1.50

Dual-sequence	2.37 +/- 1.14	1.19 +/- 0.49	2.01 +/- 0.48

## Conclusions

Both methods generated MBF estimates that are within literature ranges. However, the values between the two methods differed significantly. The AUC of the main bolus was lower than that of the dose corrected pre-bolus. This would be consistent with a reduced dose ratio, perhaps due to dilution of the main bolus in the line, or inaccuracies in the concentration conversion procedure, due to the limitations of the imaging sequence (e.g. an imperfect saturation and/or readout pulses). These results call into question the accuracy of these techniques and highlight the need for future studies to isolate the causes of these discrepancies.

## Funding

During this work S Plein was funded by a British Heart Foundation fellowship (FS/10/62/28409). S Plein received an educational research grant from Philips Healthcare. David Broadbent was funded by an NIHR fellowship NIHR-DRF-2012-005-155.

